# Feasibility and Patient Experience of a Home-Based Rehabilitation Program Driven by a Tablet App and Mobility Monitoring for Patients After a Total Hip Arthroplasty

**DOI:** 10.2196/10342

**Published:** 2019-01-31

**Authors:** Jildou Hoogland, Annet Wijnen, Tjerk Munsterman, Carina LE Gerritsma, Baukje Dijkstra, Wierd P Zijlstra, Janneke Annegarn, Francisco Ibarra, Wiebren Zijlstra, Martin Stevens

**Affiliations:** 1 Department of Orthopedics University of Groningen University Medical Center Groningen Groningen Netherlands; 2 Department of Physiotherapy Martini Hospital Groningen Groningen Netherlands; 3 Department of Orthopedics Martini Hospital Groningen Groningen Netherlands; 4 Department of Orthopedics Medical Center Leeuwarden Leeuwarden Netherlands; 5 Department of Personal Health Philips Research Eindhoven Netherlands; 6 University of Trento Trento Italy; 7 Institute of Movement and Sport Gerontology German Sport University Cologne Cologne Germany

**Keywords:** home-based rehabilitation, mobile phone, osteoarthritis, physiotherapy, total hip arthroplasty

## Abstract

**Background:**

Recent developments in technology are promising for providing home-based exercise programs.

**Objective:**

The objective of this study was to evaluate the feasibility and patient experience of a home-based rehabilitation program after total hip arthroplasty (THA) delivered using videos on a tablet personal computer (PC) and a necklace-worn motion sensor to continuously monitor mobility-related activities.

**Methods:**

We enrolled 30 independently living patients aged 18-75 years who had undergone THA as a treatment for primary or secondary osteoarthritis (OA) between December 2015 and February 2017. Patients followed a 12-week exercise program with video instructions on a tablet PC and daily physical activity registration through a motion sensor. Patients were asked to do strengthening and walking exercises at least 5 days a week. There was weekly phone contact with a physiotherapist. Adherence and technical problems were recorded during the intervention. User evaluation was done in week 4 (T1) and at the end of the program (T2).

**Results:**

Overall, 26 patients completed the program. Average adherence for exercising 5 times a week was 92%. Reasons mentioned most often for nonadherence were vacation or a day or weekend off 25% (33/134) and work 15% (20/134). The total number of technical issues was 8. The average score on the user evaluation questionnaire (range 0-5) was 4.6 at T1 and 4.5 at T2. The highest score was for the subscale “coaching” and the lowest for the subscale “sensor.”

**Conclusions:**

A home-based rehabilitation program driven by a tablet app and mobility monitoring seems feasible for THA patients. Adherence was good and patient experience was positive. The novel technology was well accepted. When the home-based rehabilitation program proves to be effective, it could be used as an alternative to formal physiotherapy. However, further research on its effectiveness is needed.

## Introduction

Surgical treatment by means of total hip arthroplasty (THA) is most often indicated in end-stage hip osteoarthritis (OA). At present, THA is considered one of the most successful, effective, and cost-effective surgical treatments available. Hence, a total of 29,937 primary THAs were performed in the Netherlands in 2017 [1]. As in other Western countries, there is an increasing tendency in the Netherlands to perform fast-track surgery, after which people leave the hospital within a few days. The downside, however, is a risk of patients being minimally supported in their rehabilitation process during hospital admission and after discharge. At present, postoperative physiotherapy is not always covered by the basic health insurance in the Netherlands [2]. This can ultimately lead to suboptimal recovery [3]. Bandholm and Kehlet emphasized the urge for immediate and intensive postoperative physiotherapy [3]. In addition, Austin et al showed that this physiotherapy need not take place in a formal setting; a home-based program could work as well [4]. Furthermore, Austin et al showed that a home-based rehabilitation program seems to be both safe and efficacious for a majority of patients undergoing THA [4].

In this context, it is important to look at how technical innovations can be supportive of such home-based programs. Recent technological developments such as wearable sensors and tablet use with mobile internet are promising for providing home-based programs [5]. The use of objective activity monitoring with wearable sensors can potentially be helpful in strategies aimed at increasing adherence to home-based rehabilitation programs and daily activity [6]. Furthermore, a home-based program can improve adherence, which is often influenced by aspects such as lack of motivation, the effort and costs of traveling, and a preference for the privacy of the home environment [7].

The use of computers and tablets is rising among older adults in the Netherlands [6]. The ownership of tablets among seniors aged 65-75 years increased from 28% in 2012 to 60% in 2016 [8]. Although home-based rehabilitation programs may be of great importance, research is needed to optimize the programs that are supported by technology. This study, therefore, aimed to evaluate the feasibility and patient experience of a home-based rehabilitation program after THA, delivered using videos on a tablet personal computer (PC) and a necklace-worn motion sensor to continuously monitor mobility-related activities.

## Methods

### Study Design

A 6-month prospective cohort study was conducted to test the feasibility and patient experience of a home-based rehabilitation program. Patients participated in a 12-week, home-based exercise program after THA, following video instructions on a tablet PC. Physical activity was registered daily through a necklace-worn motion sensor, and patients were contacted weekly by telephone to receive coaching from a physiotherapist. The phone calls were aimed at motivating participants, discussing barriers to exercise and exercise load, and answering questions concerning guidelines in terms of movement and load after surgery. Measurements were taken preoperatively (T0) and at 4 weeks (T1), 12 weeks (T2), and 6 months postoperatively (T3). The study was approved by the Medical Ethical Committee of University Medical Center Groningen (METc2014/399).

### Study Population

We included 30 independently living patients aged 18-75 years who had undergone THA as treatment for primary or secondary OA. Patients were waiting for THA at either the Martini Hospital Groningen or the Medical Center Leeuwarden in the Netherlands. Exclusion criteria were as follows: (1) revision surgery; (2) medical conditions that disallow independent living; (3) cognitive impairment; and (4) inability to sufficiently read and understand Dutch. Patients were included from December 2015 to February 2017 and were required to sign a written informed consent form to be able to participate.

### Rehabilitation Program

The duration of the program was 12 weeks. Patients started the program within 7 days of the surgery. Patients performed exercises independently at home using the tablet PC for instructions. The program included strengthening and walking exercises based on increasing the muscle force, balance, and functionality. The exercises comprised movements that trained abductors, flexors, and extensors of the affected hip. The content of the program was based on previous research [9,10] and on guidelines from the American Association of Orthopaedic Surgeons. For the rest, the program was designed in line with the most recent guidelines from the Royal Dutch Society for Physical Therapy [11].

Patients were asked to exercise at least 5 days a week, with rest days on Thursday and Sunday. Strengthening exercises were performed 3 times a week. The instructions for the exercises were provided by videos on the tablet PC, which patients had to imitate. The sessions started with exercise bouts of 10 minutes, which progressively went up to 45 minutes during the 12 weeks of the program. The first step-in level of the program consisted of light and easy exercises. Difficulty and exercise duration were increased across levels very gradually. The exercise burden increased by adding more repetitions, more exercises, and longer training time as well as by incorporating the use of ankle weights. Instructions for walking exercises had no video and showed a descriptive message only. Patients started by walking three 5-minute blocks each day, progressing up to a total of 30-minute walking per day (see Multimedia Appendix 1 for a complete overview of the home-based rehabilitation program).

At the end of the week, patients were asked questions on the tablet PC about perceived pain and perceived intensity of the exercises. A score of self-reported intensity <4 (scale 0-10) was used as an indicator that a patient could train at a higher level. There was weekly telephone support from a physiotherapist. During this phone call, the physiotherapist and the patient evaluated the progress and agreed on whether to train at a higher level. The program consisted of 12 levels, each week intended for a level of increasing difficulty.

During the intervention, the physiotherapist made 3 home visits. On the first visit, participants received an explanation about the exercises and use of the tablet. The second and third visits were, respectively, at weeks 4 and 12 postoperatively and included physical tests and filling out questionnaires.

### Technical Apps

#### Tablet Personal Computer

Patients received exercise instructions through a tablet PC, a Dell Latitude 10 running the Windows 8 operating system. Exercise instructions were provided through a Web-based app. The app provided exercise instructions and gave participants feedback on their training performance. Exercise completion and app use were recorded to track adherence. The app was designed to be as easy as possible so that people with no tablet experience could participate. Internet connection was provided by the subjects’ own home Wi-Fi.

The physiotherapist used a coach app that showed daily registration of completion degree or interruption of exercise bouts. Answers on the evaluation questions (about pain and perceived intensity) were also shown at the end of the week. The physiotherapist was able to change the level of the exercises through this app.

#### Sensor

**Figure 1:**
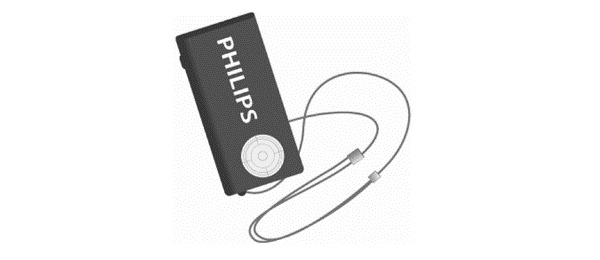
The necklace-worn motion sensor.

The necklace-worn sensor (Figure 1) weighed about 30 g and measured 55 mm × 25 mm × 10 mm (Research prototype; Philips Research, Eindhoven, the Netherlands) [6]. The sensor device included a miniature hybrid sensor containing a 3-dimensional microelectromechanical system accelerometer and a barometric pressure sensor. Accelerometry data were sampled at 50 Hz with a range of 8 g; barometric data were sampled at 25 Hz. A micro-SD card was used for storage and exchange of data. Subjects were asked to wear the sensor in the daytime during the 12-week program and connect the sensor to the tablet manually using a USB cable for data transfer and battery charging every night [6].

### Evaluation Methods

#### Patient Characteristics

Preoperative demographic data, height, weight, medical history, and pre- and postoperative complications were recorded. Factors that might have influenced patients’ ability to independently perform a home-based program using novel technology were assessed through a questionnaire. Furthermore, questions were asked about the previous and current use of PCs and smartphones.

#### Adherence

Adherence to the rehabilitation program was evaluated on the basis of the completion of the planned exercises as indicated by watching the exercise videos and reading the instruction messages. Program adherence was considered sufficient when it exceeded 70%. Reasons why patients did not perform the planned exercises were recorded by the physiotherapist during the weekly phone calls.

#### User Evaluation

User evaluation was performed with a questionnaire adapted from the sensing and action to support mobility in ambient assisted living subject evaluation form [12,13]. The questionnaire contained questions about the user experience, the perceived intensity of the intervention, coaching, wearing of the sensor, and acceptability of the technology. Answer categories ranged on a Likert scale from 0 (“Do not agree at all”) to 5 (“Fully agree”). A higher score indicated a more positive opinion. At the end of the questionnaire, patients were able to write down other suggestions or comments. The user evaluation was done at week 4 (T1) and at the end of the program (T2).

#### Technical Problems

Technical issues that interrupted the execution of the program were logged during the program. All phone calls and extra home visits were registered along with the reasons for these calls or visits.

### Statistical Analysis

Statistical analyses were performed using IBM SPSS Statistics 22.0 (IBM). Descriptive statistics were used to portray the main characteristics of the research group.

## Results

### Demographic Characteristics

In total, 9 men and 21 women participated in the study. Mean age was 64 (SD 6.7) years. Table 1 shows an overview of the demographic characteristics. Of all patients, 7 were living alone and the others were living with a partner or with partner and children. Furthermore, 8 patients had undergone THA on the other hip in the past. While 8 patients had back problems, 5 had rheumatic complaints. All patients had previous computer experience, and 25 owned a smartphone.

### Adherence rate

A total of 26 patients completed the program. Four patients dropped out in the first 2 weeks: 3 patients dropped out because of severe back pain, preference to visit a regular physical therapist, and reoperation after a fall and the fourth patient performed postoperatively worse than expected; this patient was insecure, needed more direct personal coaching, and went to a regular physical therapist. Because of sustained back pain, 2 patients finished the program 4 weeks before the official end and went to a regular physical therapist. There were no exercise-induced injuries. Of the 26 patients who completed the program, 3 did not participate in the 6-month measurement because of surgery of the other hip (THA and a fracture) and illness.

**Table 1:** Demographic characteristics of participants.

Characteristic		Participants (N=30), n (%)
Age in years, mean (SD)		64 (6.7)
**Gender, n (%)**		
	Male	9 (30)
	Female	21 (70)
Length (cm), mean (SD)		175 (7.2)
Body weight (kg), mean (SD)		79.8 (13.9)
**Education, n (%)**		
	Low	13 (43)
	Middle	4 (13)
	High	13 (43)
Employed, n (%)		17 (57)
**Living situation, n (%)**		
	Alone	7 (23)
	With partner	20 (67)
	With partner and children	2 (7)
	With children	1 (3)
**Computer experience, n (%)**		
	Daily	25 (83)
	Sometimes	5 (17)
Smartphone owners, n (%)		25 (83)
**Surgical approach, n (%)**		
	Posterolateral	22 (73)
	Anterior	8 (27)
Previous total hip arthroplasty on the other hip, n (%)		8 (27)

**Table 2:** Overview of adherence rate, self-reported perceived pain and intensity, and percentage of patients who increased a level that week during the 12-week rehabilitation program.

Week	Adherence total, mean (SD)	Adherence to strengthening exercises, mean (SD)	Adherence to walking exercises, mean (SD)	Self-reported perceived pain^a^, mean (SD)	Self-reported perceived intensity^b^, mean (SD)	Patients increasing a level (n=26), n (%)
Week 1	96.4 (9.5)	96.5 (10.4)	96.4 (13.1)	4.1 (2.0)	4.6 (2.6)	20 (77)
Week 2	96.7 (8.3)	96.3 (10.6)	97.2 (10.6)	3.6 (1.7)	5.0 (1.9)	18 (69)
Week 3	98.8 (4.3)	98.7 (6.5)	99.0 (4.9)	3.0 (1.9)	3.9 (2.1)	23 (88)
Week 4	96.9 (6.8)	97.5 (9.0)	96.2 (11.6)	2.9 (2.2)	3.0 (2.0)	20 (77)
Week 5	97.3 (6.0)	97.5 (9.0)	97.1 (8.1)	2.2 (2.3)	2.7 (2.3)	25 (96)
Week 6	96.9 (6.8)	96.2 (10.8)	98.1 (6.8)	1.9 (1.9)	2.5 (2.1)	26 (100)
Week 7	96.5 (6.9)	97.5 (9.0)	95.2 (12.3)	2.0 (1.6)	2.2 (1.9)	26 (100)
Week 8	94.6 (9.0)	93.7 (13.3)	96.2 (13.6)	1.9 (2.1)	2.1 (2.0)	22 (85)
Week 9	87.6 (22.2)	84.1 (25.6)	93 (22.3)	1.6 (1.9)	2.3 (1.8)	17 (71)^c^
Week 10	82.8 (28.4)	82.8 (30.5)	83.0 (30.4)	1.9 (1.8)	2.3 (1.8)	20 (83)^c^
Week 11	85.4 (22.5)	83.5 (24.0)	88.0 (24.9)	1.5 (1.1)	2.3 (1.8)	20 (83)^c^
Week 12	69.6 (39.6)	66.7 (40.2)	74.0 (41.6)	1.6 (1.2)	2.1 (1.9)	N/A^d^

^a^When rating perceived pain on a 0-10 scale at the end of the week (0=no pain, 10=worst possible pain).

^b^When rating perceived intensity of the exercises on a 0-10 scale at the end of the week (0=rest, 10=maximal).

^c^ Since 2 patients stopped earlier because of sustained back pain, n=24 in these weeks.

^d^N/A: not applicable.

For all patients, average adherence to exercising 5 times a week was 92%. For all weeks, the adherence was sufficient (>70%), except for strengthening exercises on week 12 (Table 2). After week 8, there was a decrease in adherence. Adherence for strengthening and walking exercises was comparable, except for weeks 9 and 12; for both these weeks, adherence to walking exercises was higher than adherence to strengthening exercises. During the intervention, self-reported perceived pain decreased from 4.1 in week 1 to 1.6 in week 12. Self-reported perceived intensity of the exercises decreased from 4.6 in week 1 to 2.1 in week 12. A score of self-reported intensity <4 was used as an indicator that a patient could train at a higher level. These results correspond with the fact that not raising the exercise level at the end of the week mostly occurred in the first 4 weeks of the program.

Table 3 shows the reasons for nonadherence. Participants failed to comply with training due to vacation or a day or weekend off 25% (33/134) of the time. In addition, work 15% (20/134) and internet connectivity problems 10% (13/134) were often mentioned as the reasons for not exercising. Holidays, days off, and work were mentioned mainly in the last 3 weeks of the intervention. Not exercising because of a social activity was mentioned on all weeks, while pain or muscle pain related to the THA was mentioned mainly in the first 2 weeks.

**Table 3:** Overview of the reasons for nonadherence.

Reasons for nonadherence	Total number of reasons (n=134), n (%)
Holiday or vacation or day or weekend off	33 (25)
Work	20 (15)
Social activity: birthday, family visit, national holiday	20 (15)
(Muscle) pain related to the total hip arthroplasty (THA)	14 (10)
Pain not related to the THA	13 (10)
Internet problems	13 (10)
Unknown	9 (7)
Forgot to do the exercises	6 (4)
App or tablet did not work	3 (2)
No motivation to train	2 (1)
Disease or illness	1 (1)

**Table 4:** Results of the user evaluation questionnaire.

Subscale^a^	Time point	
	T1^b^ (n=26), mean (SD)	T2^c^ (n=26), mean (SD)
Rehabilitation program	4.58 (0.66)	4.59 (0.65)
Coaching	4.88 (0.38)	4.85 (0.48)
Sensor	4.11 (1.00)	3.99 (1.07)
Tablet personal computer	4.77 (0.50)	4.74 (0.55)

^a^Answer options varied from “Do not agree at all” (0) to “Fully agree” (5) on a Likert scale. A higher score on the questionnaire indicated a more positive opinion on the intervention.

^b^T1: At 4 weeks into the program.

^c^T2: At the end of the program.

In this study, of the 26 patients who completed the program, 5 (19%) completed all levels of the program, 11 (42%) reached level 11, 5 (19%) reached level 10, 2 (8%) reached level 9, and 3 (12%) reached level 8 of the program. Not raising the exercise level at the end of the week occurred 43 times in total and occurred mostly in the first 2 weeks of the program. Staying on the same level occurred 23 times (23/43, 53%) in weeks 1-4 and 20 times (20/43, 47%) after week 4. Table 2 shows an overview of the increase in level each week.

The total number of technical issues was 8. Of them, 5 issues included errors in the server of the app. These problems were mostly solved within a few hours, so people were able to complete the exercises for that day. Three issues required an extra home visit to be solved—an unstable Wi-Fi connection, a broken tablet PC, and a disconnection of the sensor and the tablet PC.

### User Evaluation

The average score on the user evaluation questionnaire (range 0-5) was 4.6 at T1 and 4.5 at T2. The highest score was for the subscale “coaching” and the lowest score was for “sensor” (Table 4). For the subscale “rehabilitation program,” the highest scores were given for the statements “The rehabilitation program is effective for improving muscle strength,” “The instructions for the exercises were clear,” and “I would recommend this rehabilitation program to other patients.” Lowest scores (although >4.0) were given for the statements “The rehabilitation program is effective for improving my walking pattern” and “The level of the exercises was adapted to my possibilities.”

Overall, 19 patients gave suggestions for improvements or other comments at the end of the user evaluation questionnaire. Among all, 9 patients mentioned that they liked being able to rehabilitate from home (and that they did not have to travel) and felt motivated by the rehabilitation program; 4 patients would have liked an extra home visit in the first few weeks to check the performance of the exercises (mentioned by 2 patients) and the walking pattern (mentioned by 2 patients). Furthermore, 5 patients recommended more diversity in the exercises; 7 patients mentioned that the duration of the program was a bit too long, especially when they felt their recovery was complete and they had started working again, and 8 patients reported that they experienced the daily wearing of the sensor as uncomfortable because the sensor was big (mentioned by 4 patients) and because the cord was irritating (mentioned by 4 patients).

## Discussion

### Principal Findings

The results of this study provide support for the feasibility of a home-based telemonitored rehabilitation program for patients after a THA. Adherence to the program was good and user evaluation was positive, and there were only 8 technical issues during the intervention.

A total of 30 patients were included in the study, and 26 patients completed the program. Because of pre-existing back pain, 2 patients finished the program 4 weeks before the official end. The back pain was unrelated to the intervention. There were no exercise-induced injuries during the intervention. This indicates that patients after THA can perform a rehabilitation program safely at home. Furthermore, previous studies concluded that unsupervised home exercise is safe for a majority of THA patients [4,14].

Average adherence to exercise 5 times a week was 92%, which was higher than our goal of 70%. However, we must note that after week 8, there was a decrease. Overall, adherence rate for our program is higher than that for similar 12-week programs, such as those by Chang et al and Mikkelsen et al [14,15], who reported an adherence rate of 73% and 77%, respectively; these two home-based rehabilitation programs were not supported by technology. Our study adherence rate was comparable with the 99% rate for the 8-week home-based program combined with weekly institutional exercise sessions used by Steinhilber et al [16]. This suggests that weekly phone contact combined with the use of technology has the potential to replace supervised exercise sessions.

The reasons mentioned most often for nonadherence were vacation (or a day or weekend off) and work. Both reasons were mentioned mainly in the last 3 weeks of the intervention, which explains the decrease in adherence after week 8. Some people even suggested that the program could be shortened. Internet problems concerned 10% (13/134) of the reasons for nonadherence, although this applied only for 2 patients in a short period (6-7 days).

Patients were positive about the program, giving an average score of 4.6 (range 0-5) at T1 and 4.5 at T2 on the user evaluation questionnaire. Patients liked that they could rehabilitate from home (and that they did not have to travel) and felt motivated by the program. The remote support by weekly phone contact with the coach was appreciated by patients. The importance of the weekly phone contact is in line with a previous study reporting that motivation and coaching is an important parameter for home-based exercise performance and enhanced adherence [17].

The rehabilitation program consisted of 12 levels each week, intended for a level of increasing difficulty and exercise duration. Despite the various levels offered, 5 patients suggested more diversity in the exercises. Furthermore, 4 patients would have liked an extra home visit in the first few weeks to check performance of the exercises and their walking pattern. These comments correspond with the lowest scores in the subscale “rehabilitation program” of the evaluation questionnaire for the statements: “The rehabilitation program is effective for improving my walking pattern” and “The level of the exercises was adapted to my possibilities.” Of all, 7 patients mentioned that the program duration was a bit too long; 6 of these patients started working again 6-8 weeks postoperatively. It appears difficult to combine the program with work, even though patients could choose for themselves the time of day to exercise. A recommended adjustment is a more individualized program with additional exercise diversity and when necessary extra support, possibly in the form of a home visit, to improve the walking pattern. Another recommendation is adjusting the duration of the program to patients’ goal achievement.

Patients were positive about the technology and gave an average score of 4.8 and 4.1 (range 0-5) for the use of the tablet and sensor, respectively. All patients used their own home Wi-Fi. Geraedts et al reported that adherence to their home-based exercise program and dropping out were strongly influenced by the stability of the mobile internet connection [13]. Based on this study and that of Geraedts et al, it can be concluded that Wi-Fi is preferred over mobile internet connection. All patients had previous computer experience and most patients owned a smartphone [13]. This study shows that it is feasible for this patient group to use novel technology in a home-based rehabilitation program.

Austin et al supported unsupervised home exercise as an effective rehabilitation strategy, which is cost effective as well, for most THA patients compared with formal physiotherapy [4]. The study suggests that because of cost-effectiveness, a home-based program should be used as a standard of routine care after THA. However, some patients may benefit more from formal physiotherapy, for instance, some seniors or people with poor preoperative functional status. More research is needed to identify which patient populations benefit more from supervised rehabilitation.

A limitation of the study was the small number of patients, although this was a deliberate choice to test the feasibility of the program for the first time. In addition, patients who had agreed to participate in the study had some computer experience already and were probably more motivated than average patients, which led to some bias. Nonetheless, the wide variety in educational level, age, and living and work situation seem to have provided a representative group.

### Conclusions

A home-based rehabilitation program driven by a tablet app and mobility monitoring seems feasible for THA patients. Adherence to the program was good, and patient experience was positive. In addition, the novel technology was accepted well. When the home-based rehabilitation program also proves to be effective, it could be an alternative to formal physiotherapy. However, further research is needed into the effectiveness.

## Appendix

Multimedia Appendix 1 Content of the home-based rehabilitation program.

## Abbreviations

OA: osteoarthritis
PC: personal computer
THA: total hip arthroplasty
