# Supplementary material for: Feasibility and Patient Experience of a Home-Based Rehabilitation Program Driven by a Tablet App and Mobility Monitoring for Patients After a Total Hip Arthroplasty
Source: JMIR Mhealth Uhealth. 2019 Jan 31;7(1):e10342. doi: 10.2196/10342 (PMC6374724; doi:10.2196/10342)
Supplement: Multimedia Appendix 1 [file mhealth_v7i1e10342_app1.pdf]

Multimedia Appendix 1: Content of the home-based rehabilitation program.

	Strength exercises		Walking/Step exercise	
	(Day 1, 3 & 6)		(Day 2 & 5)	
	**Exercise**	**Intensity**	**Exercise**	**Intensity**
**Level 1**	Sitting knee extensions (operated leg)	2 x 10 reps	Walking	3x 5 min
	Standing knee raise (operated leg)	"		
	Heel and toe raises	"		
	Hip extension (operated leg)	"		
	Hip abduction (operated leg)	"		
**Level 2**	Sitting knee extensions (operated leg)	3 x 10 reps	Walking	2x 10 min
	Standing knee raise (operated leg)	"		
	Heel and toe raises	"		
	Hip extension (operated leg)	"		
	Hip abduction (operated leg)	"		
	Bilateral mini-squat behind a chair	2 x 10 reps		
**Level 3**	Heel and toe raises	3 x 10 reps	Walking	2x 10 min
	Sitting knee extensions (operated leg)	"		
	Standing knee raise (operated leg)	"	Step	2x 10 reps
	Hip extension (operated leg)	"		
	Hip abduction (operated leg)	"		
	Bilateral mini-squat behind a chair	3 x 10 reps		
**Level 4**	Heel and toe raises	3 x 10 reps	Walking	3x 10 min
	Sitting knee extensions (operated leg)	2 x 10 reps + ankle weight (1/2 kilo)		
	Standing knee raise (operated leg)	"	Step	2x 10 reps
	Hip extension (operated leg)	"		
	Hip abduction (operated leg)	"		
	Bilateral mini-squat behind a chair	3 x 10 reps		
**Level 5**	Heel and toe raises	3 x 10 reps	Walking	3x 10 min
	Sitting knee extensions (operated leg)	3 x 10 reps + ankle weight (1/2 kilo)		
	Standing knee raise (operated leg)	"	Step	3x 10 reps
	Hip extension (operated leg)	"		
	Hip abduction (operated leg)	"		
	Bilateral mini-squat behind a chair	3 x 10 reps		
**Level 6**	Tandem stance, one hand for support	2 x 10 sec	Walking	2x 15 min
	Heel and toe raises	3 x 10 reps		
	Chair rise/sit to stand	2 x 5 reps	Step	3x 10 reps
	Standing knee raise (operated leg)	3 x 10 reps + ankle weight (1/2 kilo)		
	Hip extension (operated leg)	"		
	Hip abduction (operated leg)	"		
**Level 7**	Tandem stance, one hand for support	2 x 15 sec	Walking	2x 15 min
	Heel and toe raises	3 x 10 reps		
	Chair rise/sit to stand	3 x 5 reps	Step	2x 15 reps
	Standing knee raise (operated leg)	3 x 10 reps + ankle weight (1/2 kilo)		
	Hip extension (operated leg)	"		
	Hip abduction (operated leg)	"		
	Hip abduction (non-operated leg)	"		
**Level 8**	Tandem stance, one hand for support	2 x 15 sec	Walking	2x 20 min
	Heel and toe raises	3 x 10 reps		
	Chair rise/sit to stand	3 x 5 reps	Step	2x 15 reps
	Single-leg stance, one hand for support	2 x 10 sec		
	Standing knee raise (operated leg)	3 x 15 reps + ankle weight (1/2 kilo)		
	Hip extension (operated leg)	"		
	Hip abduction (operated leg)	"		
	Hip abduction (non-operated leg)	"		
**Level 9**	Tandem stance, one hand for support	2 x 20 sec	Walking	2x 20 min
	Heel and toe raises	3 x 10 reps		
	Chair rise/sit to stand	3 x 5 reps	Step	3x 15 reps
	Single-leg stance, one hand for support	4 x 10 sec		

	Standing knee raise (operated leg)	3 x 15 reps + ankle weight (1 kilo)		
	Hip extension (operated leg)	"		
	Hip abduction (operated leg)	"		
	Hip abduction (non-operated leg)	"		
**Level 10**	Tandem stance, without hand support	2 x 20 sec	Walking	1x 25 min
	Heel and toe raises	3 x 10 reps		
	Chair rise/sit to stand	3 x 5 reps	Step	3x 15 reps
	Single-leg stance, one hand for support	4 x 15 sec		
	Standing knee raise (operated leg)	3 x 15 reps + ankle weight (1 kilo)		
	Hip extension (operated leg)	"		
	Hip abduction (operated leg)	"		
	Hip abduction (non-operated leg)	"		
**Level 11**	Tandem stance, without hand support	2 x 25 sec	Walking	1x 25 min
	Heel and toe raises	3 x 10 reps		
	Chair rise/sit to stand	3 x 5 reps	Step	4x 15 reps
	Single-leg stance, without hand support	4 x 15 sec		
	Standing knee raise (operated leg)	4 x 15 reps + ankle weight (1 kilo)		
	Hip extension (operated leg)	"		
	Hip abduction (operated leg)	"		
	Hip abduction (non-operated leg)	"		
**Level 12**	Tandem stance, without hand support	2 x 25 sec	Walking	1x 30 min
	Heel and toe raises	3 x 10 reps		
	Chair rise/sit to stand	4 x 5 reps	Step	4x 15 reps
	Single-leg stance, without hand support	4 x 20 sec		
	Standing knee raise (operated leg)	4 x 15 reps + ankle weight (1 kilo)		
	Hip extension (operated leg)	"		
	Hip abduction (operated leg)	"		
	Hip abduction (non-operated leg)	"		

Abbreviations: minutes (min); seconds (sec); repetitions (reps).
